# The Effect of Fe_3_O_4_ Nanoparticle Size on Electrical Properties of Nanofluid Impregnated Paper and Trapping Analysis

**DOI:** 10.3390/molecules25163566

**Published:** 2020-08-06

**Authors:** Bin Du, Qian Liu, Yu Shi, Yushun Zhao

**Affiliations:** School of Electrical Engineering & Automation, Hefei University of Technology, Hefei 230009, China; hf65518@163.com (Q.L.); hf45578@163.com (Y.S.); yushunzhao@126.com (Y.Z.)

**Keywords:** impregnated paper, breakdown voltage, relative permittivity, dielectric loss, trap depth

## Abstract

This paper systematically studies the effect of Fe_3_O_4_ nanoparticle size on the insulation performance of nanofluid impregnated paper. Three kinds of Fe_3_O_4_ nanoparticles with different sizes and their nanofluid impregnated papers were prepared. Environmental scanning electron microscopy (ESEM) and infrared spectroscopy were used to analyze the combination of Fe_3_O_4_ nanoparticles and nanofluid impregnated paper. The effect of nanoparticle size on breakdown voltage and several dielectric characteristics, e.g., permittivity, dielectric loss, of the nanofluid impregnated paper were comparatively investigated. Studies show that the Fe_3_O_4_ nanoparticles were bound to impregnated paper fibers by O–H bonds, while the relative permittivity and dielectric loss of the nanofluid impregnated papers were increased. Meanwhile, the increase of trap depth, caused by the nanoparticles, can trap the electric charge and improve the breakdown strength. The test results show that the direct current (DC) and alternating current (AC) breakdown voltages of nanofluid impregnated paper increased by 9.1% and 10.0% compared to FR3 nanofluid impregnated paper, respectively.

## 1. Introduction

Oil-paper insulation composed of insulating oil and oil impregnated paper is the main insulation method for electrical equipment [1,2,3]. With the improvement of the voltage level and the miniaturization of electrical equipment, the electric field intensity of oil-paper insulation increases continuously, and the corresponding insulation performance requirements become higher. It is generally believed that further improvement of the oil-paper insulation performance is the key to reducing the insulation failure rate of electrical equipment.

At present, the research on material performance improvement is a hot issue [4,5,6,7], Movahedi found that the mechanical and tribological behavior of Ni(Al) was reinforced by Al_2_O_3_–13% TiO_2_ nanoparticles [8]. A large number of studies have proved that dispersing nanoparticles into insulating oil can effectively improve the breakdown voltages and thermal conductivity of the insulating oil. JC Lee et al. found that Fe_3_O_4_ nanoparticles can increase the AC breakdown voltage of insulating oil from 12 kV/cm to 40 kV/cm [9]. Publication [10] reported that Fe_3_O_4_ nanoparticles can significantly reduce the development speed of streamer propagation under lighting voltage. The velocity of streamer propagation in insulating oil is 2.08 km/s, while the velocity of streamer propagation in Fe_3_O_4_ nanofluids is only 0.97 km/s. However, the insulating oil in a transformer can be replaced regularly, the service life of power equipment is critically determined by the oil impregnated paper [11,12]. Therefore, how to improve the insulation performance of oil impregnated paper is a key factor in improving the properties and to prolong the service life of power equipment.

Publication [13] studied the interaction between nanoparticles and insulating paper, the study found nanoparticles combined with the paper fibers and stayed inside the impregnated paper during the impregnation process, which will have an important influence on the electrical properties of the impregnated paper. Publication [14] observed the combination of nanoparticles and insulating paper through scanning electron microscope (SEM) and found that the DC breakdown voltage and AC breakdown voltage of nanofluid impregnated paper were both higher than that of insulating oil impregnated paper. Publication [15] predicted the dielectric properties of nanocellulose-modified press-paper by the multivariate analysis method. However, comparative and systematic studies on the influence of nanoparticle size on the breakdown voltages and the dielectric properties of nanofluid impregnated paper is still an open issue, which is becoming increasingly important when applying such nanofluid impregnated paper in large power equipment.

This paper aims to explore how the size of Fe_3_O_4_ nanoparticles generates the various insulation performances of nanofluid impregnated paper. Three differently sized Fe_3_O_4_ nanoparticles and their nanofluid impregnated paper were prepared. The breakdown voltages and dielectric properties of the nanofluid impregnated paper are presented and discussed, and electron trapping measurements of the impregnation were used to analyze the changes of insulation performance of the nanofluid impregnated paper.

## 2. Results and Discussion

**Figure 1:**
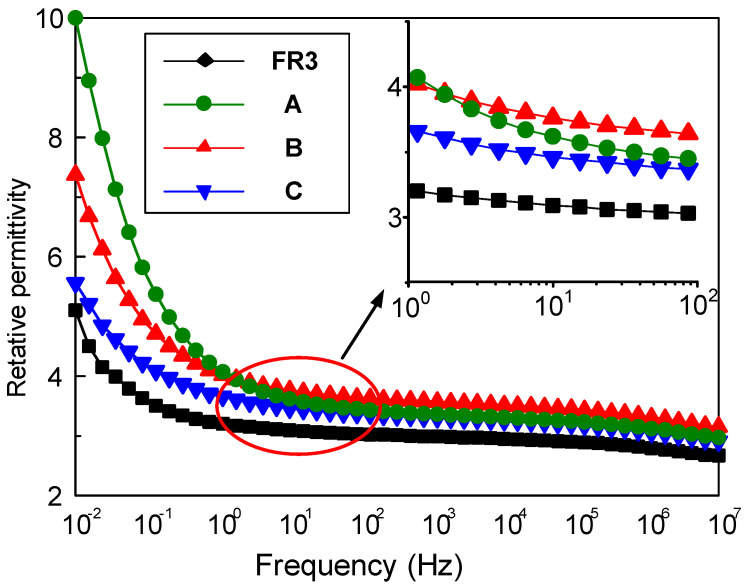
The relative permittivity of FR3 oil impregnated paper and the three different sized nanofluid impregnated papers.

The relative permittivity and dielectric loss of nanofluid impregnated paper were measured by the broadband dielectric spectrum (Concept 80, Novocontrol Technologies GmbH &Co, Cologne, Germany). Figure 1 shows the frequency dependence between the relative permittivity for FR3 impregnated paper and the three nanofluid impregnated papers. It can be seen that the relative permittivity of the nanofluid impregnated paper is significantly higher than that of FR3 oil. At 50 Hz, the relative permittivity of FR3 oil impregnated paper is 3.2, while the relative permittivity of sample A is 3.5. As is well known, the relative permittivity of Fe_3_O_4_ nanoparticles is ca. 80 [16], which is much greater than that of insulating paper, and this causes the relative permittivity increase for nanofluid impregnated paper.

One can observe that the sized nanoparticles endow nanofluid impregnated paper with varied permittivity values. According to Busessem’s analysis [17], the smaller sized nanoparticles usually possess higher permittivity than the larger ones, because in the process of the growth of nanoparticles, the energy distribution will cause internal stress changes and this results in a change of relative permittivity of the nanoparticles. The relative permittivity of nanoparticles decreases with nanoparticle size increase, and further reduces the relative permittivity of nanofluid impregnated paper.

**Figure 2:**
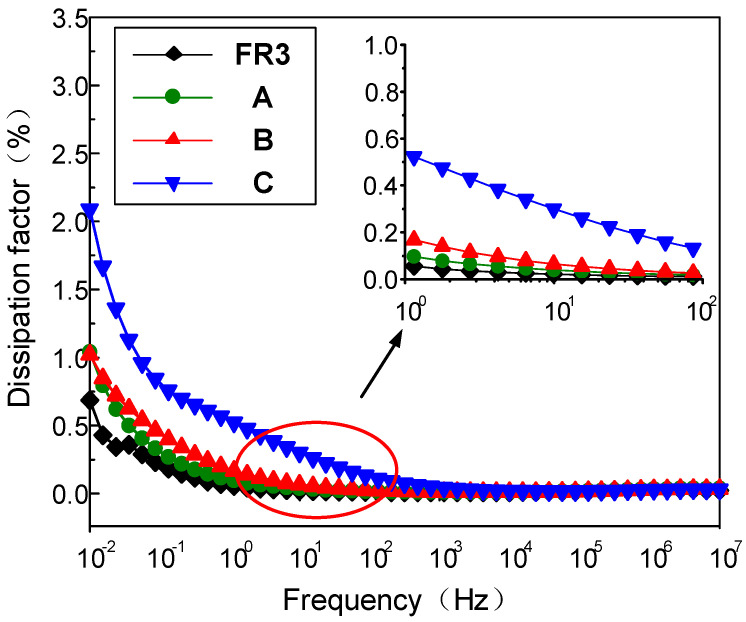
The dielectric loss of FR3 oil impregnated paper and the three nanofluid impregnated papers.

Figure 2 gives the frequency dependence of the dielectric loss of FR3 oil impregnated paper and the three nanofluid impregnated papers at different frequencies. The results show that the dielectric loss of the three different sized nanofluid impregnated papers is significantly higher than that of the FR3 oil impregnated paper in the low frequency range (<1 Hz). At the same time, the dielectric loss factor of the nanofluid impregnated paper significantly increases with the Fe_3_O_4_ nanoparticle size increase. Under the action of electric field, the dielectric loss is determined by conductance loss and polarization loss, if the dielectric conductivity is very small and so can be ignored, the dielectric loss factor can be expressed as:

(1) $$\tan\delta =\frac{\left(\varepsilon_{s}-\varepsilon_{\infty }\right)\omega \tau }{\left(\varepsilon_{s}+\varepsilon_{\infty }\right){\left(\omega \tau \right)}^{2}}$$

where *ε_s_* is the relative permittivity of the Fe_3_O_4_ nanoparticle when the frequency is zero, and *ε*_∞_ is the relative permittivity of the Fe_3_O_4_ nanoparticle when the frequency is infinite; *ω* is the angular frequency, *τ* is the polarization relaxation time of the Fe_3_O_4_ nanoparticle.

From Equation (1), it can be seen when $\omega \tau =\sqrt{\varepsilon_{s}/\varepsilon_{\infty }}\approx 1$, the value of tan*δ* is at the maximum value. Polarization loss is inversely proportional to frequency and decreases with increasing frequency. Therefore, in the frequency range of 10^−2^ to 1 Hz, the dielectric loss of nanofluid impregnated paper is significantly higher than that of FR3 oil impregnated paper.

During the growth of the nanoparticles, a large number of structural defects and holes are inevitably generated in the Fe_3_O_4_ nanoparticles, appearing more for larger sized nanoparticles. The electrical conductivity of Fe_3_O_4_ nanoparticles increases with size increase, and the high conductivity of Fe_3_O_4_ nanoparticles certainly leads to a much-increased dielectric loss for nanofluid impregned paper.

**Figure 3:**
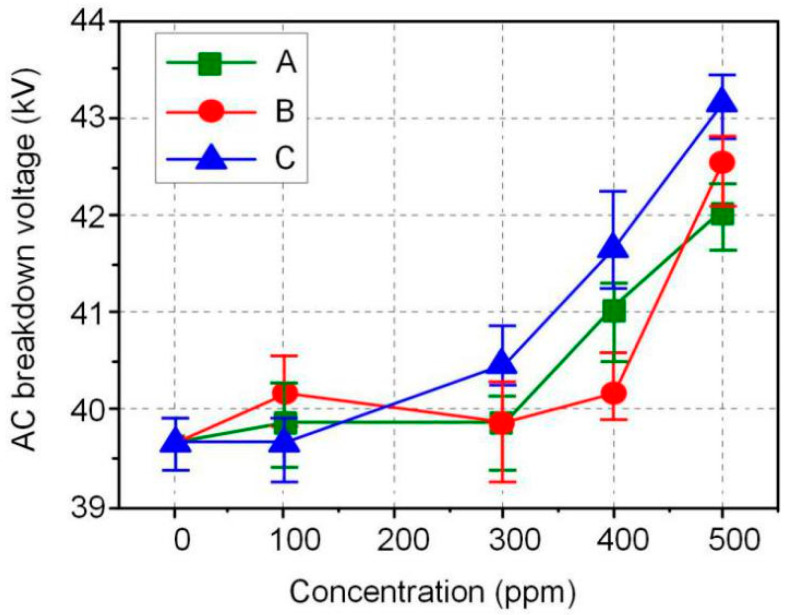
AC breakdown voltage of the different sized nanofluid impregnated papers.

**Figure 4:**
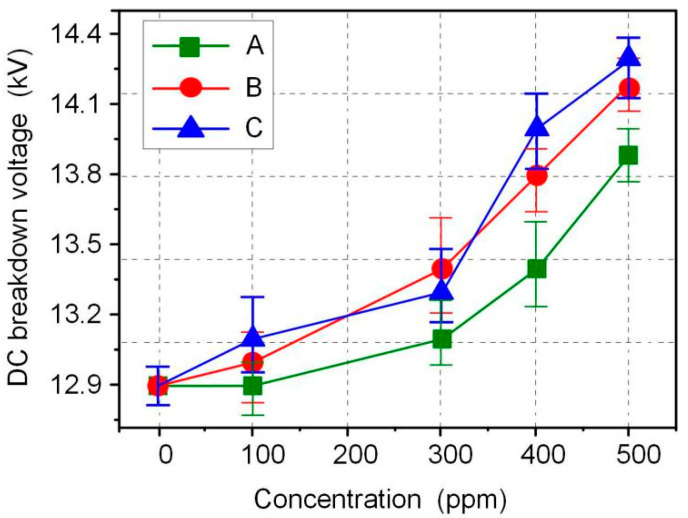
DC breakdown voltage of the different sized nanofluid impregnated papers.

The AC and DC breakdown voltages of nanofluid impregnated paper samples were measured in accordance with ASTM D149 and D3755, and the results are shown in Figure 3 and Figure 4. FR3 oil impregnated paper is recorded as an Fe_3_O_4_ nanoparticle 0 ppm nanofluid impregnated paper sample. The results show that Fe_3_O_4_ nanoparticles can significantly increase the breakdown voltage of nanofluid impregnated paper. The maximum AC breakdown voltage of Fe_3_O_4_ nanofluid impregnated paper is 43.2 kV, which is 9.1% higher than that of FR3 oil impregnated paper. The maximum DC breakdown voltage of Fe_3_O_4_ nanofluid impregnated paper samples is 14.2 kV, which is also 10.0% higher than the DC breakdown voltage of FR3 oil impregnated paper. Comparing the AC and DC breakdown voltage of the impregnated paper samples, it can be found that within a certain range, the increase of Fe_3_O_4_ nanoparticle size will increase the breakdown voltage of the nanofluid impregnated paper samples.

**Figure 5:**
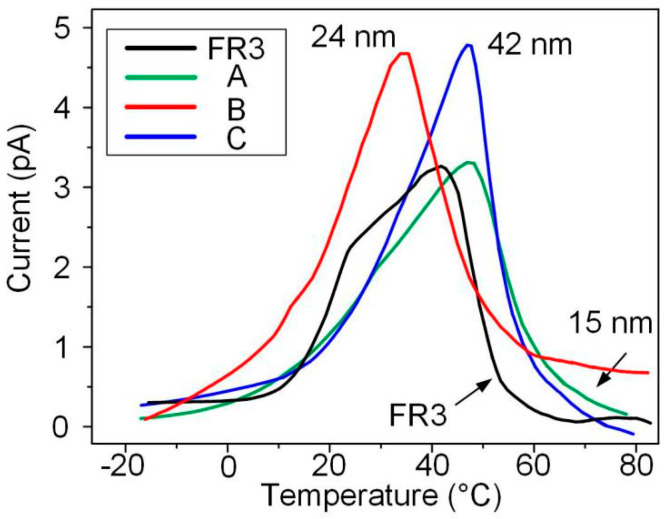
The results of thermally stimulated currents of the different impregnated papers.

Thermal stimulation current tests of nanofluid impregnated paper and FR3 nanofluids were carried out, the trapping parameters of different oil impregnated paper samples were obtained, as shown in Figure 5. 

According to publication [18], the trap levels of the oil impregnated paper samples were calculated. The formula for calculating the trap level is given by Equation (2), and the calculation results are shown in Table 1.

(2) $$E=A\frac{kT_{p}T_{i}}{T_{p}-T_{i}}$$

where *I*_p_ and *T*_p_ are the current and temperature corresponding to the peak of the the thermally stimulated current (TSC) curve, and *I*_i_ and *T*_i_ are the current and temperature corresponding to any point on the TSC curve. *A* can be expressed as:

(3) $$A=a_{1}+\sum_{n=0}\left(1-a_{2}^{n+2}e^{-A}\right)\frac{(n+1)!}{A^{n}}a_{3}^{n}{\left(-1\right)}^{n}$$

and the $a_{1}\equiv \ln(I_{p}/I_{i})$, $a_{2}\equiv T_{i}/T_{p}$, $a_{3}\equiv \Delta T/T_{i}$ and $\Delta T=T_{p}-T_{i}$.

It can be seen from Table 1 that the trap depth of nanofluid impregnated paper is greater than that of FR3 nanofluid impregnated. This indicates that the combination of Fe_3_O_4_ and paper fibers can increase the trap depth. It also can be observed that the trap depth of nanofluid impregnated paper increases as the nanoparticles size increases.

## 3. Experimental and Characterization

**Figure 6:**
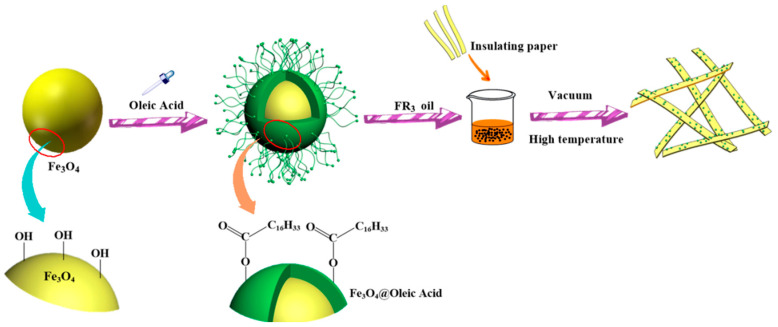
Preparation process of nanofluid impregnated paper.

The process of preparation of nanofluid impregnated paper is shown in Figure 6. The nanofluid impregnated paper was obtained via three main procedures: synthesis of Fe_3_O_4_ nanoparticles, synthesis of Fe_3_O_4_ nanofluids, and preparation of impregnated papers.

### 3.1. Fe_3_O_4_ Nanoparticles

6.48 g of ferric chloride hexahydrate, 21.9 g of sodium oleate, 48 mL of ethanol and 84 mL of n-hexane were mixed in a three-necked flask. The solution was continuously stirred for 6 h at 60 °C in a water bath, and the upper liquid after standing was retained. To the obtained solution was added 2.1 g of iron oleate, 10 mL of octadecene, 0.64 mL of oleic acid. In a nitrogen atmosphere, the mixture solution was heated to 200 °C for 2 h, with continued increase of temperature to 320 °C for 24, 48, and 72 h, respectively to realize Fe_3_O_4_ nanoparticles with varied sizes. After cooling down to room temperature, the nanoparticles were subsequently centrifuged and washed several times with ethanol and cyclohexane before drying in air at 70 °C. 

### 3.2. Fe_3_O_4_ Nanofluids

The three Fe_3_O_4_ nanoparticles obtained at different reaction times were dispersed into FR3 oil by ultrasonic treatment for 30 min. The ^®^FR3 oil was used as received [19]. Three nanofluids and the FR3 oil were dried at 85 °C under 50 Pa for 72 h to remove moisture.

### 3.3. Impregnated Paper

Insulating paper was cut into 5 × 10cm strips, then the insulating paper strips were dried under 50 Pa at 90 °C for 72 h. After drying, the insulating paper strips were removed in FR3 oil and the three kinds of nanofluids, and immersed at 70 °C for 48 h under 50 Pa. Then the FR3 insulating oil impregnated paper and the three kinds of nanofluid impregnated paper were obtained. The three nanofluid impregnated papers were tagged as sample A, B, and C, respectively.

**Figure 7:**
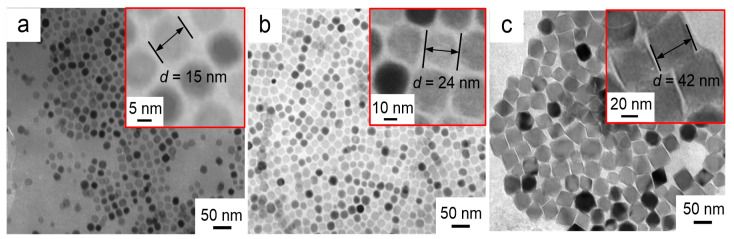
TEM images of nanoparticles at different reaction times (**a**) 24 h (**b**) 48 h (**c**) 72 h.

The morphologies of the three different nanofluid impregnated papers prepared by adding different Fe_3_O_4_ nanoparticle were characterized by transmission electron microscopy (TEM, JEM-2100F, Japan Electronics Ltd., Tokyo, Japan), and the results are shown in Figure 7a–c. The high magnification TEM images (inset in top right) clearly show the Fe_3_O_4_ nanoparticle size. It can be seen that the prepared nanoparticles are monodispersed nearly spherical particles, and each nanoparticle is composed of two distinct regions. The darker core is composed of Fe_3_O_4_ crystals, and the surrounding layer is a low-density shell of oleic acid surfactant. Covalent binding between oleic acid and Fe_3_O_4_ crystals prevents agglomeration of the Fe_3_O_4_ nanoparticles. 

The average size of the Fe_3_O_4_ nanoparticle in Figure 7a is estimated as ~15 nm. With longer reaction time, i.e., from 24 to 72 h, three different sized Fe_3_O_4_ nanoparticles were obtained with varying diameter from ~15 (Figure 7a) to ~42 nm (Figure 7c) via ~24 nm (Figure 2B). 

**Figure 8:**
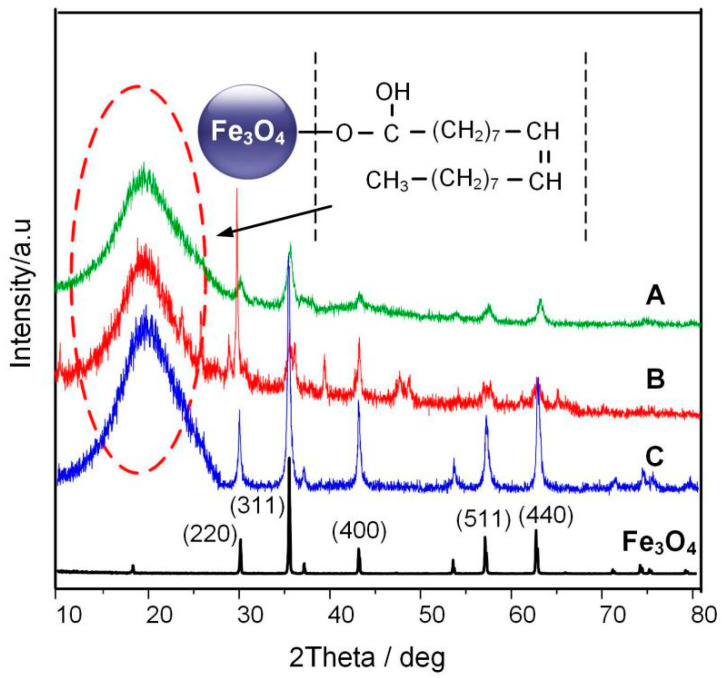
XRD patterns of pure Fe_3_O_4_ and Fe_3_O_4_ nanoparticle at different reaction times.

The X-ray diffraction (XRD) pattern was obtained by using a powder X-ray diffraction meter equipped with a rotating anode and a Cu-Kα radiation source. The scan step was 0.02°. Figure 8 shows the XRD spectrum of a typical Fe_3_O_4_ nanoparticle according to [20] and the three Fe_3_O_4_ nanoparticles prepared by the different reaction times. According to JCPDS card number 65-3107, the 2θ values of 30.1°, 35.5°, 43.1°, 56.9°, and 62.6° are signatures of (220), (311), (400), (511), and (440) crystal face for Fe_3_O_4_ nanoparticle, respectively. At the same time, it can be seen from the figure that Fe_3_O_4_ nanoparticles show a huge amorphous peak near 20°, of the oleic acid surfactant coated on the surface of Fe_3_O_4_, this peak represents the oleic acid surfactant coated on the Fe_3_O_4_ nanoparticle.

**Figure 9:**
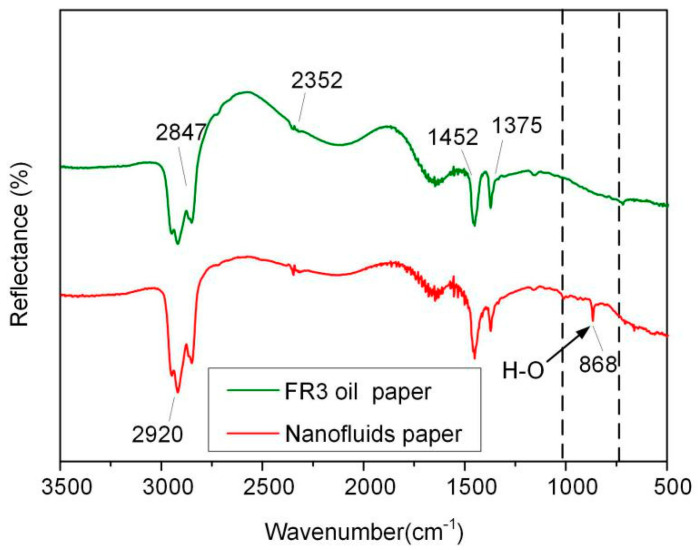
ATR-FTIR of FR3 oil impregnated paper and nanofluid impregnated paper.

In order to further analyze the bonding between Fe_3_O_4_ nanoparticles and paper fibers, the FR3 impregnated paper and nanofluid impregnated paper were tested by attenuated total reflection Fourier infrared spectroscopy (ATR-FTIR, Nicolet, Thermo Electron Corporation, Franklin, TN, USA), and the results are shown in Figure 9. In the spectrum, symmetric and asymmetric vibration peaks of –CH_2_ and –CH bands appear at 2847, 2920, 1452, 1375 cm^−1^, respectively, and the stretching vibration absorption of C=O band at 2352 cm^−1^ appears in the spectrum. These indicate the presence of insulating oil molecules or oleic acid in the paper fiber. It is worth noting that, a new absorption band of an O–H group 868 cm^−1^ appears in the nanofluid impregnated paper, indicating the bonding of Fe_3_O_4_ nanoparticle onto the surfaces of the paper fiber.

**Figure 10:**
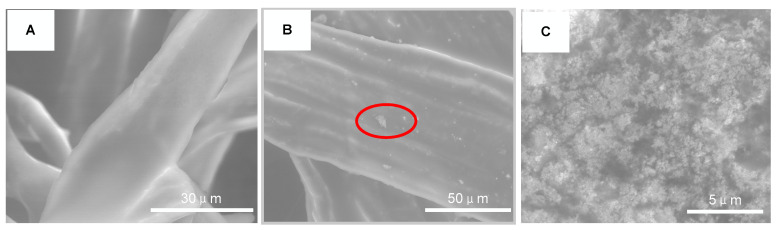
ESEM images of FR3 oil impregnated paper (**A**) nanofluids impregnated paper (**B**) and high magnification image of nanofluids impregnated paper marking area (**C**).

The surface morphology of FR3 oil impregnated paper and Fe_3_O_4_ nanofluid impregnated paper were observed by ESEM (Quattro S, Thermo Fisher Scientific Inc., Waltham, MA, USA), as shown in Figure 10. It can be seen that the surface of the FR3 nanofluid is smooth without any significant particle protrusions. However, a large number of nanoparticles can be observed on the surface of the Fe_3_O_4_ nanofluid impregnated paper. When the image is magnified to 16,000 times, as shown in Figure 5C, it can be clearly seen that the nanoparticles are agglomerated to each other and bonded tightly to the paper fiber.

## 4. Conclusions

Fe_3_O_4_ nanoparticles and insulating paper fibers were combined through O–H bond linkage to form nanofluid impregnated paper. The AC and DC breakdown voltages of nanofluid impregnated paper are 9.1% and 10.0% higher than that of FR3 oil impregnated paper, respectively. In a certain range, the increase of Fe_3_O_4_ nanoparticles size will be beneficial to improve the insulation performance of the nanofluid impregnated paper.

The relative dielectric constant and dielectric loss of nanofluid impregnation are both higher than that of FR3 oil impregnated paper. When the Fe_3_O_4_ nanoparticle size is 42 nm and test frequency is 50 Hz, the relative dielectric constant and dielectric loss for nanofluid impregnated paper are 3.5 and 0.3%. Meanwhile, with Fe_3_O_4_ size increase, the relative dielectric constant of nanofluid impregnated paper decreases, and the dielectric loss increases.

The TSC results show that the Fe_3_O_4_ nanoparticles will significantly increase the trap depth of nanofluid impregnated paper, and the trap depth increases with the increase of nanoparticle size. The increase in the depth of the trap makes it more difficult for free charges to escape the bondage of the trap, thereby increasing the insulation performance of the nanofluid impregnated paper.

## Figures and Tables

**Table 1:** TSC peak current and trap depth of the impregnated papers.

Sample	Peak Current (pA)	Trap Depth (eV)	Standard Deviation (eV)
FR3 oil impregnated paper	3.25	0.543	0.026
Nanofluid impregnated paper A	3.37	0.551	0.053
Nanofluid impregnated paper B	4.66	0.640	0.118
Nanofluid impregnated paper C	4.78	0.772	0.093

## References

[1] Li J., He Z., Bao L., Yang L. (2011). Influences of Corrosive Sulfur on Copper Wires and Oil-Paper Insulation in Transformers. Energies.

[2] Wang S.-Q., Zhang G.-J., Mu H.-B. (2012). Effects of Paper-aged State on Space Charge Characteristics in Oil-impregnated Paper Insulation. IEEE Trans. Dielectr. Electr. Insul..

[3] Pradhan A., Chatterjee B., Chakravorti S. (2014). Effect of temperature on frequency dependent dielectric parameters of oil-paper insulation under non-sinusoidal excitation. IEEE Trans. Dielectr. Electr. Insul..

[4] Marsavina L., Sadowski T., Faur N. (2011). Numerical investigation of the stress field near a crack normal to ceramic-metal interface. J. Mech. Sci. Technol..

[5] Guo J., Wang X., Jia Z., Wang J., Chen C. (2018). Nonlinear Electrical Properties and Field Dependency of BST and Nano-ZnO-Doped Silicone Rubber Composites. Molecules.

[6] Marsavina L., Constantinescu D.M., Linul E., Apostol D.A., Voiconi T., Sadowski T. (2014). Refinements on fracture toughness of PUR foams. Eng. Fract. Mech..

[7] Serban D.A., Weber G., Marsavina L., Vadim V., Silberschmidt V.V., Hufenbach W. (2013). Tensile properties of semi-crystalline thermoplastic polymers: Effects of temperature and strain rates. Polym. Test..

[8] Movahedi B. (2014). Mechanical and Tribological Behavior of Ni(Al)-Reinforced Nanocomposite Plasma Spray Coatings. J. Therm. Spray Technol..

[9] Kamruzzaman Selim K.M., Ha Y.S., Kim S.J., Chang Y., Kim T.J., Lee G.H., Kang I.K. (2007). Surface modification of magnetite nanoparticles using lactobionic acid and their interaction with hepatocytes. Biomaterials.

[10] Li J., Zhang Z.-T., Zou P., Du D., Liao R.-J. (2012). Lightning impulse breakdown characteristics and electrodynamic process of insulating vegetable oil-Based nanofluid. Mod. Phys. Lett. B.

[11] Wei Y.-H., Zhu M.-X., Li Y., Zhao L., Deng J.-B., Mu H.-B., Zhang G.-J. (2015). Partial discharge characteristics and trap parameters of aged oil-impregnated paper. IEEE Trans. Dielectr. Electr. Insul..

[12] Okabe S., Koto M., Nara T., Suganuma K., Takahashi K. (1997). Deterioration characteristics of power capacitors with oil-impregnated paper or film. Electr. Eng. Jpn..

[13] Segal V., Rabinovich A., Nattrass D. (2000). Experimental study of magnetic colloidal fluids behavior in power transformers. J. Magn. Magn. Mater..

[14] Zhou Y., Zhong Y.-x., Chen M.-t., Zhang S.-n., Du Y.-f., Lv Y.-z., Li C., Liu T. (2012). Effect of nanoparticles on electrical characteristics of transformer oil-based nanofluids impregnated pressboard. IEEE Trans. Dielectr. Electr. Insul..

[15] Zhou Y., Huang X., Huang J., Zhang L., Zhou Z. (2018). Predicting the Dielectric Properties of Nanocellulose-Modified Presspaper Based on the Multivariate Analysis Method. Molecules.

[16] Hwang J.G., Zahn M., O’Sullivan F.M., Pettersson L.A.A., Hjortstam O., Liu R. (2010). Effects of nanoparticle charging on streamer development in transformer oil-based nanofluids. J. Appl. Phys..

[17] Buessem W.R., Cross L.E., Goswami A.K. (1966). Phenomenological Theory of High Permittivity in Fine-Grained Barium Titanate. J. Am. Ceram. Soc..

[18] Lei Q.-q., Wang X., Fan Y. (1992). A new method of auto-separating thermally stimulated current. J. Appl. Phys..

[19] Liu Q., Wang Z.D. (2011). Streamer characteristic and breakdown in synthetic and natural ester transformer liquids under standard lightning impulse voltage. IEEE Trans. Dielectr. Electr. Insul..

[20] Li J., Zhang Z., Zou P., Stanislaw G., Zahn M. (2012). Preparation of a vegetable oil-based nanofluid and investigation of its breakdown and dielectric properties. IEEE Electr. Insul. Mag..

